# Correction: Omar et al. Antifungal Evaluation and Molecular Docking Studies of *Olea europaea* Leaf Extract, *Thymus* *vulgaris* and *Boswellia carteri* Essential Oil as Prospective Fungal Inhibitor Candidates. *Molecules* 2021, *26*, 6118

**DOI:** 10.3390/molecules27123794

**Published:** 2022-06-13

**Authors:** Hanaa S. Omar, Soheir N. Abd El-Rahman, Sheikha M. AlGhannam, Nour El-Houda A. Reyad, Mohamed S. Sedeek

**Affiliations:** 1 Department of Genetics, Faculty of Agriculture, Cairo University, Giza 12613, Egypt; 2 GMO Laboratory, Faculty of Agriculture, Cairo University, Research Park, CURP, Giza 12613, Egypt; 3 Crops Technology Research Department, Food Technology Research Institute, Agricultural Research Center, Giza 12619, Egypt; 4 Department of Chemistry, College of Science, Imam Abdulrahman Bin Faisal University, P.O. Box 1982, Dammam 31441, Saudi Arabia; sm_ghannam@yahoo.com; 5 Plant Pathology Department, Faculty of Agriculture, Cairo University, Giza 12613, Egypt; nourelhoudareyad53@gmail.com; 6 Pharmacognosy Department, Faculty of Pharmacy, Cairo University, Giza 11562, Egypt; mohamed.sedeek@pharma.cu.edu.eg

The authors of this paper [1] have agreed that they would like to add Nour El-Houda A. Reyad as a co-author, as she contributed to the results of the morphological characterization and provided the initial source of *Fusarium oxysporum f. sp. lactucae*. The *Fusarium oxysporum f. sp. lactucae* is the scientific name of the studied fungus. Therefore, it must be written according to the scientific rules. Therefore, the style of this scientific name has to be corrected throughout the whole manuscript. The E-mail address of Mohamed S. Sedeek that was sent previously is now corrected to mohamed.sedeek@pharma.cu.edu.eg. The previous E-mail address used by Hanaa is now replaced by the official one hanaa.omar@agr.cu.edu.eg, as very recently required by Cairo University. This would positively reflect the actual citation record of our university accordingly. The authors apologize for any inconvenience caused and state that the scientific conclusions are unaffected. The original publication has also been updated.
